# Surgical outcomes associated with partial upper sternotomy in obese aortic disease patients

**DOI:** 10.1186/s13019-022-01890-y

**Published:** 2022-05-31

**Authors:** Zeng-Rong Luo, Yi-Xing Chen, Liang-wan Chen

**Affiliations:** 1 Department of Cardiovascular Surgery and Cardiac Disease Center, Union Hospital, Fujian Medical University, Fuzhou, 350001 People’s Republic of China; 2 Department of Cardiology, Nan Ping First Hospital Affiliated to Fujian Medical University, Nanping, 353000 People’s Republic of China

**Keywords:** Aortic, Obese, BMI, Minimally invasive, Partial upper sternotomy

## Abstract

**Background:**

Excellent partial upper sternotomy outcomes have been reported for patients undergoing aortic surgery, but whether this approach is particularly beneficial to obese patients remains to be established. This study was developed to explore the outcomes of aortic surgical procedures conducted via a partial upper sternotomy or a full median sternotomy approach in obese patients.

**Methods:**

We retrospectively examined consecutive acute type A aortic dissection patients who underwent aortic surgery in our hospital between January 2015 to January 2021. Patients were divided into two groups based on body mass index: ‘non-obese’ and ‘obese’. We then further stratified patients in the obese and non-obese groups into partial upper sternotomy and full median sternotomy groups, with outcomes between these two sternotomy groups then being compared within and between these two body mass index groups.

**Results:**

In total, records for 493 patients that had undergone aortic surgery were retrospectively reviewed, leading to the identification of 158 consecutive obese patients and 335 non-obese patients. Overall, 88 and 70 obese patients underwent full median sternotomy and partial upper sternotomy, respectively, while 180 and 155 non-obese patients underwent these respective procedures. There were no differences between the full median sternotomy and partial upper sternotomy groups within either BMI cohort with respect to preoperative baseline indicators and postoperative complications. Among non-obese individuals, the partial upper sternotomy approach was associated with reduced ventilation time (*P* = 0.003), shorter intensive care unit stay (*P* = 0.017), shorter duration of hospitalization (*P* = 0.001), and decreased transfusion requirements (Packed red blood cells: *P* < 0.001; Fresh frozen plasma: *P* < 0.001). Comparable findings were also evident among obese patients.

**Conclusions:**

Obese aortic disease patients exhibited beneficial outcomes similar to those achieved for non-obese patients via a partial upper sternotomy approach which was associated with significant reductions in the duration of intensive care unit residency, duration of hospitalization, ventilator use, and transfusion requirements. This surgical approach should thus be offered to aortic disease patients irrespective of their body mass index.

## Introduction

Aortic root aneurysm and aortic dissection are extremely serious vascular emergencies associated with high morbidity and mortality rates [1, 2]. Appropriate approaches to the management of aortic arch aneurysm patients are still being developed and optimized [3]. The conventional surgical approach for these patients is full median sternotomy (FMS), although minimally invasive partial upper sternotomy (PUS) has been employed as an alternative approach since the 1990s [4], even in obese patients [5]. However, FMS remains the standard approach for complex aortic surgery to ensure appropriate exposure and safety [6]. Even so, recent studies have explored minimally invasive surgical approaches to accessing the aortic root [7–9], ascending aorta [10], or aortic arch [3, 11–13]. These authors have reported successful surgical outcomes without increasing mortality or major complications. However, few studies have compared outcomes in obese patients with aortic disease following treatment via a PUS approach or conventional FMS.

As such, this study was designed to compare aortic disease patient outcomes between obese and non-obese patients that underwent treatment via a PUS to those of patients that were treated using a conventional FMS approach.

## Patients and methods

Following approval from the Ethics Committee of Fujian Medical University Union Hospital, China (Approval No.: 2014KY038; Date: July 25, 2014), the records of patients treated from January 2015 to January 2021 were reviewed. We received written informed consent from subjects or their legal representatives before study initiation.

### Patient groups

**Fig. 1:**
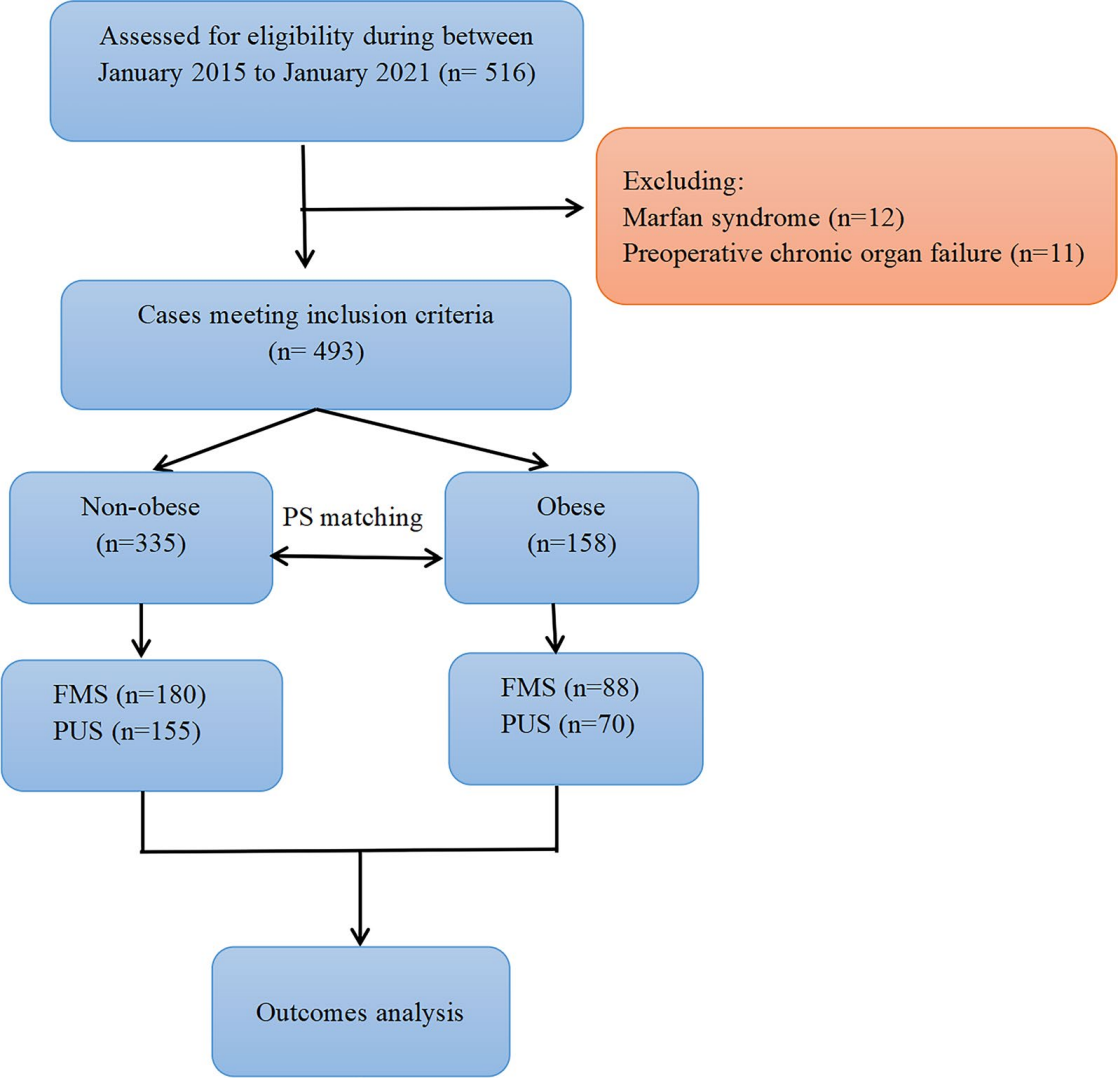
The participant selection process

Patients were divided into two groups based on body mass index (BMI): ‘non-obese’ (BMI < 27.5 kg/m^2^) and ‘obese’ (BMI ≧ 27.5 kg/m^2^). The BMI thresholds selected to define obesity were based on the BMI criteria established by the World Health Organization for Asian populations [14]. We then divided the patients in the obese and non-obese groups into partial upper sternotomy and full median sternotomy groups based on the surgical approaches employed in their treatment. Patients were excluded if they: (1) had been diagnosed with aortitis, Marfan syndrome, metoxoarteritis, or systemic immune disorders, or (2) had previously undergone organ transplantation or experienced infective endocarditis, cardiogenic shock, malignancies, or chronic organ failure. The participant selection process is outlined in Fig. 1.

### Study endpoints

Preoperative characteristics, operative parameters, and postoperative outcomes were compared between the FMS and PUS subgroups in the obese and non-obese cohorts. PUS was performed at the discretion of the operating surgeon through December 2016. Beginning in January 2017, PUS was the standard approach for all aortic surgical procedures. No additional preoperative assessments were performed for patients undergoing PUS. The expertise of the operating surgeons was similar for all patients, with all procedures having been performed by well-trained surgeons.

A pain chart with a maximum level of 10 was used to assess pain levels in unmedicated patients once per day after the patient is fully awake, with nursing staff recording the results.

### Surgical techniques

#### Minimally invasive aortic surgery via a PUS approach

**Fig. 2:**
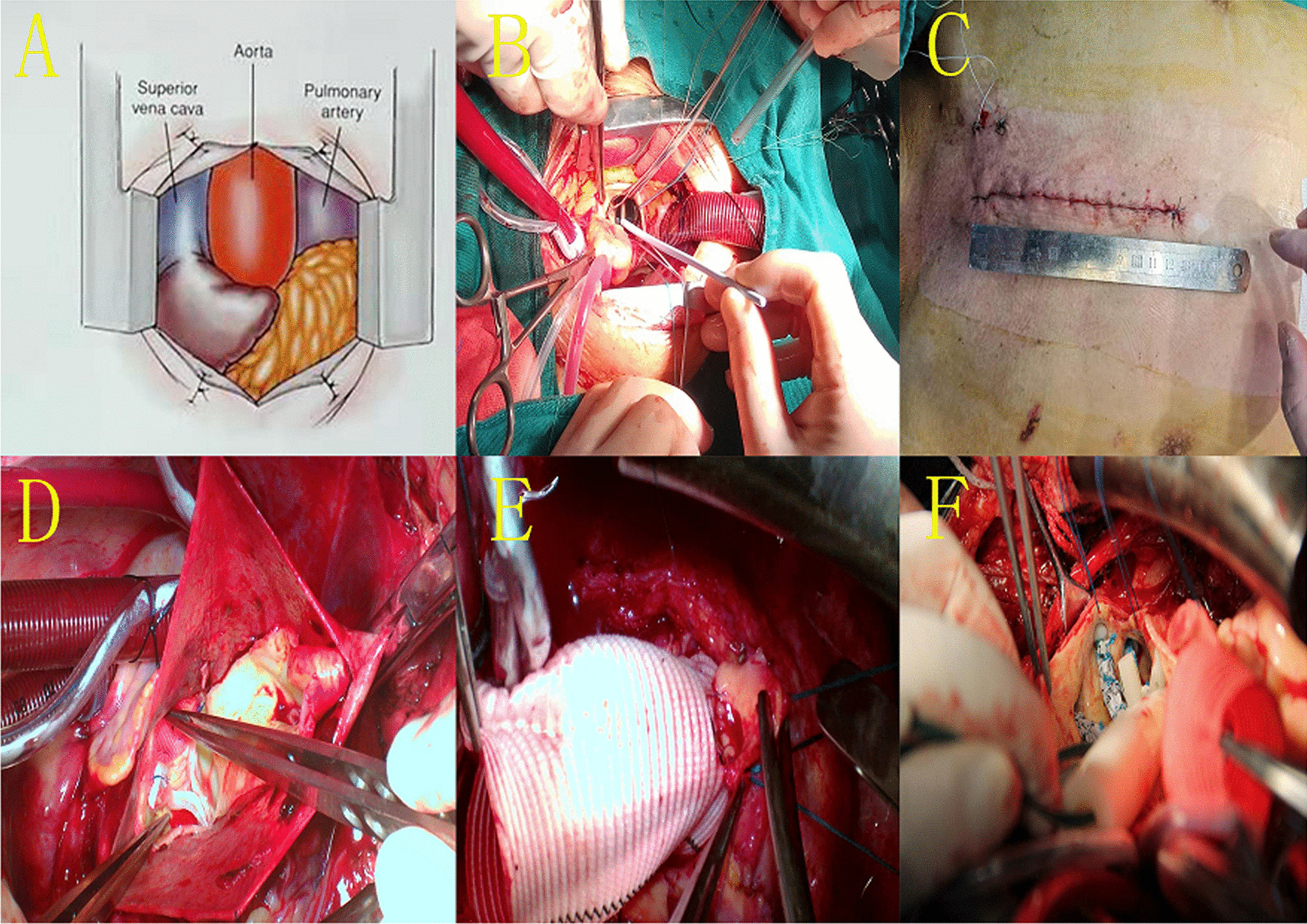
Partial upper sternotomy (PUS) approach (**A**–**C**); Aortic root reconstruction: **D** Procedure of valve-sparing aortic root replacement (VSARR), **E** Procedure of Bentall; **F** Implant and release the modified triple-branched stent-graft (MTBSG)

An 8–12 cm cutaneous incision was made, after which the sternum was incised in a J-form manner from the sternal notch to the right fourth intercostal space (Fig. 2A–C).

#### Aortic root reconstruction approach

A valve-sparing aortic root replacement (VSARR) procedure involved a modified David technique and a patch neointima technique [15] was used to repair the aortic valve in patients with severe AR, as detailed previously (Fig. 2D) [15, 16].

The Bentall procedure was defined as the replacement of the aortic root with a composite valve-graft device [17] (Fig. 2E).

#### Total arch and descending aortic replacement approach

Procedure details pertaining to the implantation of a modified triple-branched stent-graft (MTBSG) have been described previously [18]. Briefly, the branching arteries in the aortic arch were exposed, after which a MTBSG was implanted and released (Fig. 2F).

### Statistical analysis

The Shapiro–Wilk test was used to assess data distributions. Continuous data are given as means ± standard deviations, and normally distributed data were compared between groups using t-tests. Categorical variables are given as percentages and were compared with chi-squared or Fisher’s exact tests, as appropriate. *P* < 0.05 was the significance threshold, and SPSS 26.0 (SPSS Inc.) was used for all analyses.

## Results

### Data

The Shapiro–Wilk test was applied to assess the distributions of all continuous variables, and all of these variables were found to conform to a normal distribution.

### Patient characteristics

In total, 335 consecutive non-obese and 158 consecutive obese patients were identified. Overall, 88 and 70 obese patients underwent FMS and PUS, respectively, while 180 and 155 non-obese patients underwent these respective procedures. Similar risk profiles were observed when comparing these obese and non-obese patient cohorts with respect to their individual comorbidities (Table 1).

**Table 1:** Preoperative characteristics

	Total	Non-obese		P1	Obese		P2	P3	P4
		FMS	PUS		FMS	PUS			
Patients (n)	493	180 (36.5)	155 (31.5)	–	88 (17.8)	70 (14.2)	–		
Age (years)	53.9 ± 12.5	54.4 ± 10.4	52.7 ± 9.8	0.126	55.9 ± 10.8	54.0 ± 11.5	0.287	0.275	0.384
BMI (kg/m^2^)	26.99 ± 5.48	25.4 ± 3.9	24.8 ± 4.0	0.166	30.11 ± 3.04	30.56 ± 3.09	0.360	< 0.001	< 0.001
Male, n (%)	393 (79.7)	149 (82.8)	119 (76.8)	0.171	70 (79.5)	55 (78.6)	0.881	0.520	0.766
Chronic diseases									
Diabetes, n (%)	81 (16.4)	28 (15.6)	25 (16.1)	0.886	16 (18.2)	12 (17.1)	0.865	0.586	0.849
Hypertension, n (%)	402 (81.5)	144 (80.0)	126 (81.3)	0.766	79 (89.8)	63 (90.0)	0.962	0.044	0.099
Hyperlipidemia, n (%)	85 (17.2)	27 (15.0)	23 (14.8)	0.967	20 (22.7)	15 (21.4)	0.845	0.118	0.222
Coronary heart disease, n (%)	40 (8.1)	14 (7.8)	14 (9.0)	0.679	8 (9.1)	4 (5.7)	0.426	0.713	0.396
Renal dysfunction^a^, n (%)	112 (22.7)	40 (22.2)	33 (21.3)	0.837	20 (22.7)	19 (27.1)	0.523	0.926	0.335
COPD, n (%)	29 (5.9)	8 (4.4)	8 (5.2)	0.759	7 (8.0)	6 (8.6)	0.889	0.373	0.495
OSAS, n (%)	48 (9.7)	15 (8.3)	14 (9.0)	0.821	9 (10.2)	10 (14.3)	0.436	0.610	0.237
Moderate or severe AR, n (%)	170 (34.5)	60 (33.3)	53 (34.2)	0.868	30 (34.1)	27 (38.6)	0.560	0.902	0.525
Malperfusion syndromes, n (%)	88 (17.8)	32 (17.8)	29 (18.7)	0.826	15 (17.0)	12 (17.1)	0.987	0.882	0.778
EF, (%)	62.7 ± 6.7	63.5 ± 9.9	62.8 ± 7.7	0.468	61.7 ± 8.9	62.5 ± 10.2	0.600	0.150	0.827
Serum creatinine (umol/L)	118.8 ± 98.6	112.6 ± 87.8	113.9 ± 98.4	0.898	119.6 ± 98.6	120.5 ± 88.5	0.953	0.557	0.632
Hb (mg/dl)	12.58 ± 2.05	12.56 ± 2.35	12.86 ± 2.84	0.298	12.66 ± 2.88	12.69 ± 2.01	0.939	0.778	0.608
HCT (%)	41.50 ± 3.96	40.98 ± 3.96	41.80 ± 3.64	0.051	40.94 ± 3.87	41.57 ± 3.88	0.312	0.938	0.260
Primary indication				0.422			0.742	0.893	0.209
Aortic aneurysm	83 (16.8)	33 (18.3)	22 (14.2)		14 (15.9)	14 (20.0)			
Type A aortic dissection	401 (81.4)	144 (80.0)	128 (82.6)		73 (83.0)	56 (80.0)			
Type A AIH	9 (1.8)	3 (1.7)	5 (3.2)		1 (1.1)	0 (0.0)			

Continuous variables are confirmed normally distributed and are expressed as mean ± SD, categorical variables are expressed as number (%). Chi-square or Fisher test for categorical variables and t test for continuous variables

P1, P2: *P* value of FMS group versus PUS group in non-obese and obese patients, respectively

P3, P4: *P* value of non-obese patients versus obese patients in FMS and PUS group, respectively

*AR* aortic valve regurgitation, *LVEF* left ventricular ejection fraction, *COPD* chronic obstructive pulmonary disease, *OSAS* obstructive sleep apnoea syndrome, *EF* ejection fraction, *Hb* haemoglobin, *HCT* haematocrit, *SD* standard deviation, *AIH* aortic intramural hematoma

^a^Defined as preoperative creatinine greater than 1.5 mg/dL

### Operative characteristics

There were no significant differences in total operative duration, cardiopulmonary bypass time, SCP, or low body arrest when comparing surgical approaches in the obese and non-obese patient cohorts (Table 2), although the mean cross-clamp time was significantly longer in the PUS group for both non-obese (55.8 ± 26.9 min vs. 48.8 ± 17.8 min; *P* = 0.006) and obese patients (56.0 ± 19.8 min vs. 49.0 ± 16.5 min; *P* = 0.017).

**Table 2:** Procedural data

	Total	Non-obese		P1	Obese		P2	P3	P4
		FMS	PUS		FMS	PUS			
Patients, n (%)	493	180 (36.5)	155 (31.5)	–	88 (17.8)	70 (14.2)	–		
Catogeries of surgery, n (%)									
ASA + hemi-arch	54 (11.1)	18 (10.0)	18 (11.6)	0.635	10 (11.4)	8 (11.4)	0.990	0.732	0.968
ASA + total arch	52 (10.6)	18 (10.0)	17 (11.0)	0.773	9 (10.2)	8 (11.4)	0.809	0.954	0.919
Root + ASA	80 (16.2)	29 (16.1)	24 (15.5)	0.875	15 (17.0)	12 (17.1)	0.987	0.846	0.753
Root + ASA + hemi-arch	140 (28.3)	49 (27.2)	45 (29.0)	0.713	26 (29.5)	20 (28.6)	0.894	0.691	0.944
Root + ASA + total arch	167 (33.8)	66 (36.7)	51 (32.9)	0.471	28 (31.8)	22 (31.4)	0.958	0.435	0.827
Type of procedure, n (%)									
VSARR	281 (57.0)	104 (57.8)	91 (58.7)	0.912	46 (52.3)	40 (57.1)	0.630	0.433	0.884
Bentall	106 (21.5)	40 (22.2)	29 (18.7)	0.498	23 (26.1)	14 (20.0)	0.450	0.540	0.855
VSARR or Bentall + MTBSG	219 (44.4)	84 (46.7)	68 (43.9)	0.660	37 (42.0)	30 (42.9)	1.000	0.515	1.000
Operation time (min)	290.5 ± 87.5	288.5 ± 97.8	292.8 ± 100.8	0.693	290.9 ± 99.8	295.8 ± 105.8	0.766	0.851	0.839
Cardiopulmonary bypass (min)	139.8 ± 35.8	138.4 ± 43.9	142.6 ± 41.8	0.373	139.9 ± 45.6	144.4 ± 50.6	0.558	0.796	0.795
Cross-clamp time (min)	48.9 ± 18.7	48.8 ± 17.8	55.8 ± 26.9	0.006	49.0 ± 16.5	56.0 ± 19.8	0.017	0.930	0.950
SCP and low body arrest (min)	14.1 ± 4.1	13.8 ± 4.8	14.5 ± 6.9	0.290	14.1 ± 8.8	14.8 ± 7.7	0.601	0.766	0.771

Continuous variables are confirmed normally distributed and are expressed as mean ± SD, categorical variables are expressed as number (%). Chi-square or Fisher test for categorical variables and t test for continuous variables

P1, P2: *P* value of FMS group versus PUS group in non-obese and obese patients, respectively

P3, P4: *P* value of non-obese patients versus obese patients in FMS and PUS group, respectively

*ASA* ascending aorta, *SCP* selective cerebral perfusion, *VSARR* valve-sparing aortic root replacement, *MTBSG* modified triple-branched stent-graft, *SD* standard deviation

### Postoperative outcomes

For full details regarding the postoperative outcomes for patients included in this study, see Table 3. Rates of deep surgical site infection (DSSI) requiring revision, re-exploration, postoperative myocardial infarction, neurological dysfunction, renal dysfunction, hepatic insufficiency, pulmonary complications, and in-hospital mortality were also comparable between FMS and PUS subgroups in both the obese and non-obese cohorts. Neurological dysfunction was defined by delayed awakening, disorientation, convulsions, hemiplegia, severe limb muscle dysfunction, or coma. Renal dysfunction was defined as a 50% rise in baseline creatinine levels or a new need for dialysis. Hepatic insufficiency was defined as a bilirubin level greater than 5 mg/dL persisting for more than 5 days postoperatively. Pneumonia was defined by a chest roentgenogram-based diagnosis of pneumonia after cardiac surgery.

**Table 3:** Postoperative event rates of clinical outcomes

Events	Total	Non-obese			Obese			P3	P4
		FMS	PUS	P1	FMS	PUS	P2		
Patients, n (%)	493	180 (36.5)	155 (31.5)	–	88 (17.8)	70 (14.2)	–		
Infections, n (%)									
SSI	38 (7.7)	11 (6.1)	13 (8.4)	0.421	7 (8.0)	7 (10.0)	0.653	0.571	0.694
DSSI	18 (3.7)	5 (2.8)	5 (3.2)	1.000	4 (4.5)	4 (5.7)	1.000	0.694	0.607
Cardiac, n (%)									
Resternotomy for major bleeding	21 (4.3)	7 (3.9)	6 (3.9)	0.983	4 (4.5)	4 (5.7)	1.000	1.000	0.786
Cardiac arrest	15 (3.0)	5 (2.8)	4 (2.6)	1.000	3 (3.4)	3 (4.3)	1.000	1.000	0.789
MI	10 (2.0)	3 (1.7)	3 (1.9)	1.000	2 (2.3)	2 (2.9)	1.000	1.000	1.000
Neurologic dysfunction^a^	18 (3.7)	6 (3.3)	6 (3.9)	1.000	3 (3.4)	3 (4.3)	1.000	1.000	1.000
Temporary	12 (2.4)	4 (2.2)	4 (2.6)	1.000	2 (2.3)	2 (2.9)	1.000	1.000	1.000
Permanent	6 (1.0)	2 (1.1)	2 (1.3)	1.000	1 (1.1)	1 (1.4)	1.000	1.000	1.000
Renal, n (%)									
Acute kidney injury^b^	157 (31.8)	57 (31.7)	55 (35.5)	0.460	25 (28.4)	20 (28.6)	0.982	0.587	0.309
Dialysis (%)	107 (21.7)	37 (20.6)	32 (20.6)	0.984	20 (22.7)	18 (25.7)	0.663	0.683	0.397
Hepatic insufficiency^c^	145 (29.4)	50 (27.8)	46 (29.7)	0.701	26 (29.5)	23 (32.9)	0.655	0.763	0.632
Pulmonary, n (%)									
Pneumonia^d^	335 (68.0)	125 (69.4)	92 (59.4)	0.054	70 (79.5)	48 (68.6)	0.115	0.081	0.187
Reintubation	97 (19.7)	36 (20.0)	28 (18.1)	0.653	19 (21.6)	14 (20.0)	0.525	0.762	0.730
Tracheotomy	67 (13.6)	24 (13.3)	20 (12.9)	0.907	14 (15.9)	9 (14.3)	0.589	0.570	0.992
Ventilation time (h)	108.2 ± 82.3	107.2 ± 62.2	90.2 ± 40.6	0.003	129.8 ± 77.8	106.2 ± 60.0	0.033	0.019	0.045
Multiple organ dysfunction syndrome^e^, n (%)	16 (3.2)	6 (3.3)	5 (3.2)	0.956	2 (2.3)	3 (4.3)	0.794	0.923	0.993
Transfusion requirements									
Packed red blood cells (units)	5.77 ± 4.96	6.75 ± 4.73	4.34 ± 2.99	< 0.001	6.88 ± 4.66	4.76 ± 3.08	0.001	0.832	0.335
Fresh frozen plasma (mL)	470.8 ± 150.8	480.6 ± 188.6	410.0 ± 99.6	< 0.001	511.9 ± 174.9	435.8 ± 108.5	0.001	0.193	0.082
Platelets (units)	9.18 ± 5.50	9.66 ± 6.50	8.96 ± 6.70	0.333	10.03 ± 5.32	8.88 ± 5.56	0.188	0.620	0.926
Length of stay									
ICU (days)	5.8 ± 3.7	6.3 ± 3.5	5.5 ± 2.6	0.017	7.8 ± 4.7	5.5 ± 3.5	0.001	0.080	1.000
Hospital (days)	18.9 ± 14.8	20.0 ± 10.8	16.2 ± 9.8	0.001	21.5 ± 10.5	17.2 ± 9.9	0.010	0.282	0.481
In-hospital mortality, n (%)	25 (5.1)	10 (5.6)	7 (4.5)	0.666	5 (5.7)	3 (4.3)	0.974	1.000	1.000

Continuous variables are confirmed normally distributed and are expressed as mean ± SD, categorical variables are expressed as number (%). Chi-square or Fisher test for categorical variables and t test for continuous variables

P1, P2: *P* value of FMS group versus PUS group in non-obese and obese patients, respectively

P3, P4: *P* value of non-obese patients versus obese patients in FMS and PUS group, respectively

*DSSI* deep surgical site infection requiring revision, *MI* myocardial infarction, *SSI* surgical site infection

^a^Defined as coma, delayed awakening, disorientation, convulsions, hemiplegia, severe limb muscle dysfunction, etc.

^b^Defined as 50% rise in baseline creatinine or new need for dialysis

^c^Defined as bilirubin greater than 5 mg/dL persisting for more than 5 days postoperatively

^d^Defined as chest roentgenogram diagnosing pneumonia after cardiac surgery

^e^Defined as two or more organs or systems simultaneously or sequentially in the process of acute diseases such as severe trauma, shock, infection, and major surgical operations

Among obese patients, PUS treatment was associated with decreases in ventilation time [106.2 ± 60.0 vs. 129.8 ± 77.8 h; *P* = 0.033], ICU stay length [5.5 ± 3.5 days vs. 7.8 ± 4.7 days; *P* = 0.001], hospitalization duration [17.2 ± 9.9 days vs. 21.5 ± 10.5 days; *P* = 0.010], and transfusion requirements (Packed red blood cells: 4.76 ± 3.08 units vs. 6.88 ± 4.66 units, *P* = 0.001; Fresh frozen plasma: 435.8 ± 108.5 ml vs. 511.9 ± 174.9 ml, *P* = 0.001). Similar trends were also evident for non-obese patients.

**Fig. 3:**
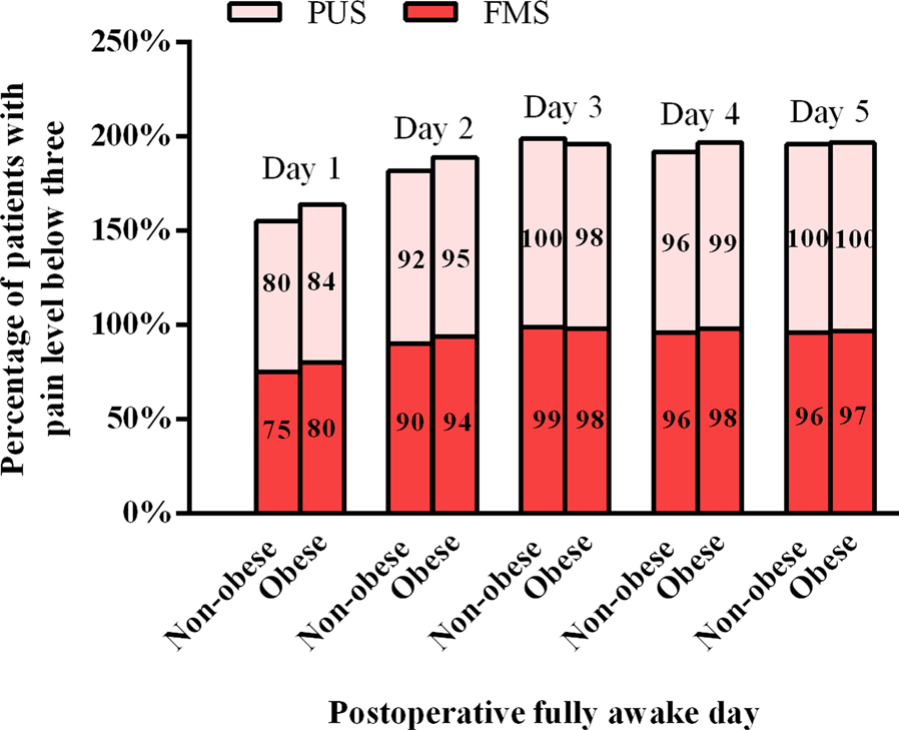
The percentage of patients reporting a pain level below 3 on a scale with a maximum level of 10 in obese and non-obese patients after fully awake

The postoperative daily (Day1–Day5) percentages of fully awake patients who reported either free of pain or experienced only minor pain (reporting a pain level below 3 on a scale with a maximum level of 10) were similar across groups (with all *P* > 0.05) (Fig. 3).

## Discussion

Surgical procedures of the ascending aorta with or without total arch replacement and aortic root reconstruction have traditionally been performed via an FMS approach to ensure sufficient exposure. Recent advances in minimally invasive surgical techniques in combination with different partial sternotomy approaches have been employed as an alternative to FMS [12, 19–22], and have been used to conduct isolated heart valve disease treatment, Bentall, hemi-arch replacement, and ascending aorta repair procedures. These less invasive approaches have been linked to superior cosmetic and postoperative outcomes, including an overall reduction in surgical trauma, ventilator use, ICU stay duration, transfusion requirements, respiratory failure, and sternal stability as compared to the FMS approach [19, 23, 24].

Although the Bentall technique and valve-sparing aortic root surgery are complex procedures that necessitate good exposure, the value of PUS as an alternative access strategy for these complex procedures has been a topic of recent interest [9–11]. Hillebrand et al. [2] evaluated outcomes for 33 patients undergoing aortic root replacement with the Bentall procedure through a J-shaped PUS access and thereby confirmed the safety of PUS when conducting complex aortic surgery. Wachter et al. [25] also demonstrated the safety of valve-sparing aortic root replacement procedures when performing the David procedure using a PUS approach.

The degree of obesity is correlated with increases in the incidence of certain adverse outcomes including renal failure, sternal and wound infections, hospitalization duration, and prolonged mechanical ventilation [26–29]. To determine whether the benefits of PUS were reduced due to patient obesity among individuals undergoing aortic surgery, we herein compared PUS and FMS outcomes for obese and non-obese patients. To the best of our knowledge, this study is the most detailed analysis of this topic to date.

### Comorbidities, operative duration, and major complication rates

In our study, we observed comparable preoperative risk profiles and operative durations for both obese and non-obese patients when comparing the PUS and FMS groups. Although PUS was associated with a longer cross-clamp duration, we do not believe that this difference, on the scale of minutes of ischemic time, is likely to be clinically relevant.

Rates of mortality and major complications were comparable in the PUS and FMS groups irrespective of BMI status, indicating that obese patients are good candidates for PUS treatment.

### Pulmonary complications

Obesity has been linked to prolonged ventilator use and increased hypoxemia after surgical procedures when treating episodes of acute aortic dissection (AAD) [30]. MIS approaches better preserve the integrity of the chest wall and thus have the potential to decrease the length of postoperative ventilator use. While obese patients did exhibit prolonged ventilator use relative to non-obese patients when comparing the PUS patient cohorts, PUS treatment was nonetheless associated with reductions in ventilator use for both obese and non-obese patients as compared to FMS treatment. We additionally observed no significant differences in rates of pneumonia, reintubation, or tracheotomy in the PUS group for obese or non-obese patients, suggesting that limited surgical access does not result in unfavorable pulmonary outcomes even among obese patients.

### Transfusion requirements

Patients in the present study that underwent treatment via a PUS approach exhibited reduced transfusion requirements as compared to patients treated via an FMS approach irrespective of whether or not they were obese. These results are in line with those of Wu et al. [31] and Xie et al. [13, 20, 32, 33]. Previous evidence suggests that transfusions are associated with a negative impact on patient outcomes following cardiac surgery [34–37]. Obese participants in the present study that underwent PUS procedures did not exhibit any differences in transfusion requirements as compared to non-obese patients, further supporting the fact that this procedure does not expose obese individuals to greater risk.

### Length of stay

MIS approaches are associated with decreased length of hospitalization and a shorter duration of ICU admittance [38]. Consistently, we found that both obese and non-obese patients in the PUS cohort exhibited shorter durations of hospitalization and ICU admittance as compared to patients in the FMS group. These findings are also consistent with previous meta-analyses [13, 23–26]. We did not observe any differences in ICU or hospital stay length for obese patients in this study relative to non-obese patients in the PUS cohort, indicating that this MIS approach is not associated with any increased risk for obese individuals.

### Sternal infections

We did not observe any protective benefits with respect to the odds of postoperative sternum infection in the PUS cohort, potentially contradicting subjective clinical expectations. This may be attributable to the fact that the pathogenesis of sternal infections is multifactorial, and as such, the improved integrity of the sternum alone is not sufficient to reduce the risk of sternum infection. Notably, we did not observe any increased risk of postoperative sternal infections among obese patients in the PUS cohort in this study, suggesting that PUS does not expose obese patients to any additional risk of sternal infection.

### Postoperative pain levels

Our results suggest that the PUS approach was associated with better postoperative pain levels. While these results do not align with those of a pooled analysis performed by Lim et al. [32], they are consistent with a meta-analysis conducted by Brown et al. [18] Overall, relatively limited data are available pertaining to this operative outcome, potentially explaining these contradictory results. We additionally found that obese and non-obese patients in the PUS cohort experienced comparable levels of postoperative pain.

### Limitations

The present study was a retrospective analysis, and it is thus inherently subject to potneital bias. In addition, the study period was relatively long, and changes in perioperative therapeutic regimens over this period may have impacted these findings. Propensity score matching could not be performed, given that the inclusion criteria for the PUS group shifted over the course of the study period from being at the discretion of the operating surgeon to the standard departmental approach.

## Conclusions

**Fig. 4:**
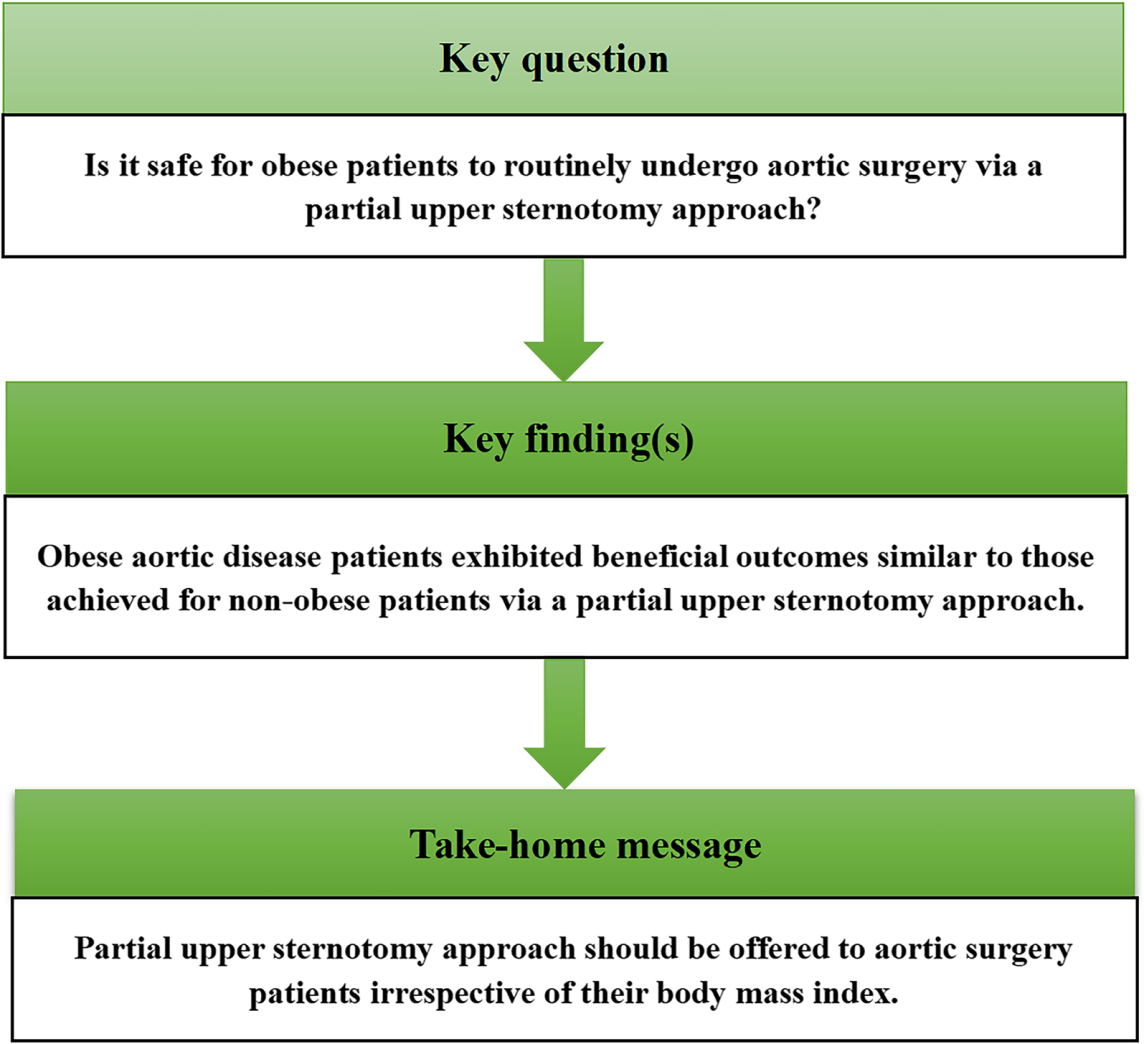
An illustrative summary of our findings

The results of this study indicate that it is both safe for obese patients to routinely undergo aortic root reconstruction or extensive total arch replacement via a PUS approach, and that this treatment approach is associated with good efficacy. This minimally invasive strategy did not adversely impact safety outcomes for obese patients, while still conferring benefits including reductions in postoperative ventilator use, ICU stay length, duration of hospitalization, and transfusion requirements. (see Fig. 4) However, further prospective randomized trials will be necessary to confirm and expand upon these findings.

## Data Availability

The data that support the findings of this study are available from Fujian Cardiac Medical Center but restrictions apply to the availability of these data, which were used under license for the current study, and so are not publicly available. Data are however available from the authors upon reasonable request and with permission of Fujian Cardiac Medical Center.

## Abbreviations

PUS: Partial upper sternotomy
FMS: Full median sternotomy
BMI: Body mass index
MIS: Minimally invasive surgery
VSARR: Valve-sparing aortic root replacement
MTBSG: Modified triple-branched stent-graft
AAD: Acute aortic dissection
ARDS: Acute respiratory disease syndrome
ICU: Intensive care unit
